# 94. Pooled analysis of nirsevimab resistance through 150 days post dose in preterm and term infants

**DOI:** 10.1093/ofid/ofac492.019

**Published:** 2022-12-15

**Authors:** Michael E Abram, Bahar Ahani, David E Tabor, Fiona Fernandes, Deidre Wilkins, Anastasia A Aksyuk, Kevin M Tuffy, Hong Ji, Christine Blaze, Tyler Brady, Pamela Griffin, Amanda Leach, Tonya L Villafana, Mark T Esser

**Affiliations:** AstraZeneca, Gaithersburg, Maryland; AstraZeneca, Gaithersburg, Maryland; AstraZeneca, Gaithersburg, Maryland; AstraZeneca, Gaithersburg, Maryland; Translational Medicine, Vaccines & Immune Therapies, BioPharmaceuticals R&D, AstraZeneca, Gaithersburg, MD, USA, Gaithersburg, Maryland; Translational Medicine, Vaccines & Immune Therapies, BioPharmaceuticals R&D, AstraZeneca, Gaithersburg, MD, USA, Gaithersburg, Maryland; AstraZeneca, Gaithersburg, Maryland; AstraZeneca, Gaithersburg, Maryland; AstraZeneca, Gaithersburg, Maryland; AstraZeneca, Gaithersburg, Maryland; AstraZeneca, Gaithersburg, Maryland; AstraZeneca, Gaithersburg, Maryland; AstraZeneca, Gaithersburg, Maryland; AstraZeneca, Gaithersburg, Maryland

## Abstract

**Background:**

Respiratory syncytial virus (RSV) is the major cause of lower respiratory tract infection (LRTI) and hospitalization in infants. In two global pivotal placebo-controlled studies, nirsevimab, a monoclonal antibody to the RSV prefusion (F) protein with extended half-life, reduced medically attended (MA) RSV LRTI versus placebo throughout the RSV season (MELODY (Primary Cohort)/Study 3 (Proposed Dose) Pool, 79.5% efficacy). Here we summarize resistance analyses of all RT-PCR-confirmed RSV isolates from healthy term and preterm infants through 150 days post dose.

**Methods:**

Infants were randomized 2:1 to receive one intramuscular injection of nirsevimab or placebo, prior to their first RSV season. RT-PCR-confirmed RSV isolates were reflexed for genotypic analyses of RSV F and phenotypic analyses of identified substitutions in a recombinant RSV neutralization susceptibility assay.

**Results:**

In the pooled proposed dose analysis of Study 3 (50 mg nirsevimab if < 5 kg at dosing) and MELODY (50 or 100 mg nirsevimab if < 5 kg or ≥5 kg at dosing, respectively), no subject with MA RSV LRTI had an RSV isolate containing nirsevimab resistance-associated substitutions in either treatment group (nirsevimab, RSV A: 0/14 and RSV B: 0/5; placebo, RSV A: 0/35 and RSV B: 0/16). In Study 3 (50 mg nirsevimab if ≥5 kg at dosing), 2/18 subjects in the nirsevimab group and 0/20 subjects in the placebo group with MA RSV LRTI had an RSV isolate harbouring nirsevimab binding site substitutions I64T+K68E+I206M+Q209R (>447-fold) or N208S (>387-fold) that conferred reduced susceptibility to nirsevimab neutralization (nirsevimab, RSV A: 0/9 and RSV B: 2/9; placebo, RSV A: 0/10 and RSV B: 0/10). Subjects with RSV isolates harboring F protein sequence variations that maintained susceptibility to nirsevimab neutralization were balanced between treatment groups with no association with RSV disease severity. No subjects with non-protocol defined MA RSV LRTI cases or hospitalization due to any RSV respiratory illness had an RSV isolate conferring nirsevimab resistance.

**Conclusion:**

Lack of nirsevimab resistance following immunization at the proposed dose supports efficacy and neutralization activity of nirsevimab against both RSV A and B strains throughout the RSV season.

**Disclosures:**

**Michael E. Abram, PhD**, AstraZeneca: Employee|AstraZeneca: Stocks/Bonds **Bahar Ahani, BSC**, AstraZeneca: Employee|AstraZeneca: Stocks/Bonds **David E. Tabor, PhD**, AstraZeneca: Employee|AstraZeneca: Stocks/Bonds **Fiona Fernandes, PhD**, AstraZeneca: Stocks/Bonds **Deidre Wilkins, BSC**, AstraZeneca: Employee|AstraZeneca: Stocks/Bonds **Anastasia A. Aksyuk, PhD**, AstraZeneca: Employee|AstraZeneca: Stocks/Bonds **Kevin M. Tuffy, MS**, AstraZeneca: Employee|AstraZeneca: Stocks/Bonds **Hong Ji, BSc**, AstraZeneca: Stocks/Bonds **Christine Blaze, BSc**, AstraZeneca: Stocks/Bonds **Tyler Brady, MS**, AstraZeneca: Employee|AstraZeneca: Stocks/Bonds **Pamela Griffin, MD**, AstraZeneca: Stocks/Bonds **Amanda Leach, MD**, AstraZeneca: Employee|AstraZeneca: Stocks/Bonds **Tonya L. Villafana, PhD, MPH**, AstraZeneca: Employee|AstraZeneca: Stocks/Bonds **Mark T. Esser, PhD**, AstraZeneca: Stocks/Bonds.

